# Expression and functional analysis of lncRNAs in the hippocampus of immature rats with status epilepticus

**DOI:** 10.1111/jcmm.14676

**Published:** 2019-11-18

**Authors:** Jing Gan, Lingyi Huang, Yi Qu, Rong Luo, Qianyun Cai, Fengyan Zhao, Dezhi Mu

**Affiliations:** ^1^ Department of Pediatrics West China Second University Hospital Sichuan University Chengdu China; ^2^ Key Laboratory of Birth Defects and Related Diseases of Women and Children (Sichuan University) Ministry of Education Sichuan University Chengdu China; ^3^ West China College of Stomatology Sichuan University Chengdu China

**Keywords:** apoptosis, ceRNA, lncRNA, miRNA, status epilepticus

## Abstract

Long non‐coding RNAs (lncRNAs) have been implicated in the regulation of gene expression at various levels. However, to date, the expression profile of lncRNAs in status epilepticus (SE) was unclear. In our study, the expression profile of lncRNAs was investigated by high‐throughput sequencing based on a lithium/pilocarpine‐induced SE model in immature rats. Furthermore, weighted correlation network analysis (WGCNA), gene ontology (GO) analysis and Kyoto Encyclopedia of Genes and Genomes (KEGG) analysis were performed to construct co‐expression networks and establish functions of the identified hub lncRNAs in SE. The functional role of a hub lncRNA (NONRATT010788.2) in SE was investigated in an in vitro model. Our results indicated that 7082 lncRNAs (3522 up‐regulated and 3560 down‐regulated), which are involved in cell proliferation, inflammatory responses, angiogenesis and autophagy, were dysregulated in the hippocampus of immature rats with SE. Additionally, WGCNA identified 667 up‐regulated hub lncRNAs in turquoise module that were involved in apoptosis, inflammatory responses and angiogenesis via regulation of HIF‐1, p53 and chemokine signalling pathways and via inflammatory mediator regulation of TRP channels. Knockdown of an identified hub lncRNA (NONRATT010788.2) inhibited neuronal apoptosis in vitro. Taken together, our study is the first to demonstrate the expression profile and potential function of lncRNAs in the hippocampus of immature rats with SE. The defined hub lncRNAs may participate in the pathogenesis of SE via lncRNA‐miRNA‐mRNA network.

## 1 INTRODUCTION

Status epilepticus (SE) is the second‐ most common neurological disorder, with an annual incidence of 10‐41 cases in a population of 100 000.^1^, ^2^ As irreversible cerebral damage occurs, it is necessary to promptly stop convulsions of SE and prevent their recurrence.^3^ The morbidity and mortality of SE are determined by the duration of epileptic activity, rapid identification of the cause of SE, and age and comorbidity of the patients.^4^ During SE development, hypoxic stress and inflammatory stress are initiated together with microglia and macrophage activation, which induce several cerebral processes resulting in neuron cell apoptosis, and further severe neuronal damage.^5^, ^6^

Long non‐coding RNAs (lncRNAs) are new members of the ncRNA family that are longer than 200 nucleotides in length.^7^ LncRNAs have been implicated in the regulation of gene expression at the epigenetic, transcriptional or post‐transcriptional level, even though they do not encode any protein products themselves.^7^, ^8^ Through binding to the specific miRNAs, lncRNA could function as a competing endogenous RNA (ceRNA) in regulating protein expression.^9^, ^10^ Various studies have revealed that lncRNAs are widely expressed in several human tissues and cells,^11^, ^12^ and participate in progression of multiple diseases, including tumorigenesis,^13^, ^14^ hepatitis C virus infection^15^ and neurobiology of stress and depression.^16^ In SE, H19—a lncRNA—was demonstrated to contribute to epileptogenesis by aggravating SE‐induced neuronal loss, glial cell activation, mossy fibre sprouting and cognitive impairments in epileptic rats.^17^ Whole‐transcriptome screening revealed that H19 exhibited diverse functions related to epileptogenesis, including demyelination, immune and inflammatory responses, cell apoptosis and activation of MAPK.^18^ UCA1, another lncRNA, may participate in the pathogenesis of epilepsy, which is evidenced by the dynamic change in the expressions of UCA1 and NF‐κB during epilepsy.^19^ However, the expression and potential function of lncRNAs in SE are still unclear.

Here, we determined the expression profile of lncRNAs by high‐throughput sequencing based on a lithium/pilocarpine‐induced SE model in immature rats. KEGG and GO analyses were performed to predict the potential function of dysregulated lncRNAs. The hub lncRNAs were determined by weighted correlation network analysis (WGCNA), and their potential function was predicted according to the lncRNA‐miRNA‐mRNA network. Furthermore, the functional role of a hub lncRNA (NONRATT010788.2) in SE was investigated in an in vitro model.

## 2 MATERIALS AND METHODS

### 2.1 SE model

The SE model was established as our previous study indicated.^20^ Briefly speaking, female Sprague Dawley rats with mixed‐sex litters were housed in a temperature‐ and light‐controlled facility with food and water. The 25‐ days‐ old pubs were intraperitoneally (i.p.) injected with lithium chloride (125 mg/kg, Sigma) at 18 hours before pilocarpine injection (i.p. injection, 40 mg/kg, Sigma). Racine's scale was performed to determine the severity of convulsions of rats, and the animals with a score of 4‐5 were used in the present study. Then, diazepam (10 mg/kg) was intraperitoneally injected to terminate the seizure attacks of the SE rats. The rats that were injected with the same amount of normal saline were defined as control. All the animals were purchased from the animal centre of Sichuan University, and related research was approved by the Sichuan University Committee on Animal Research. At 24 hours post‐SE onset, the animals (n = 4 for SE group and control group each) were killed for hippocampus dissection. The hippocampus were preserved in RNAlater (−20°C, Qiagen) for further RNA extraction.

### 2.2 LncRNA library construction and high‐throughput sequencing

The libraries preparation and deep sequencing were performed by Novogene Bioinformatics Technology Cooperation. Briefly speaking, equivalent total RNAs were used to construct the sulphur‐replete and sulphur‐deprived libraries by NEB Next^®^ Ultra™ Directional RNA Library Prep Kit for Illumina^®^ (NEB) following manufacturer's recommendations. RNA was broken into fragments by divalent cations under elevated temperature in NEBNext First Strand Synthesis Reaction Buffer and converted to first strand cDNA performed with random hexamer primer and M‐Mu LV Reverse Transcriptase. Second strand cDNA was synthesized subsequently performed with DNA Polymerase I and RNase H. dNTPs with dTTP were replaced by dUTP in the reaction buffer. Remaining overhangs were converted into blunt ends by exonuclease/polymerase. Adaptors with hairpin loop structure were ligated to prepare for hybridization after adenylation of DNA 3′ ends. For selecting 150 ~ 200 nucleotides cDNA fragments, the library was purified with AMPure XP system (Beckman Coulter) 12. Then size‐selected, adaptor‐ligated cDNA was incubated with 3 μL USER Enzyme (NEB, USA) at 37°C for 15 minutes followed by 5 minutes at 95°C. Then, PCR was performed to obtain enriched cDNA library. At last, products were purified (AMPure XP system) and assessed (Agilent Bioanalyzer 2100 system). The clustering of the index‐coded samples was performed on a cBotCluster Generation System performed with TruSeq PE Cluster Kit v3‐cBot‐HS (Illumia) according to the manufacturer's instructions. After cluster generation, sequencing of libraries was performed on the Illumina HiSeqXten platform. Reads with more than 10% N (Unable to determine base information), with adapter sequence, or of low quality were removed from the raw reads to obtain clean reads. Finally, clean reads were compared with rat genome from NCBI using hisat 2. Differentially expressed genes were selected according to the threshold set for a fold change ≥ 2.0 and a unadjusted *P* value ≤ .05. *P* values were calculated with a *t* test. All of the raw data were supplied on line (BioProject: PRJNA532235 https://dataview.ncbi.nlm.nih.gov/object/PRJNA532235?reviewer=h0ao7p9dhmmaj16jtik7nas70u). Differential expression test was analysed performed with DESeq R packages according to the packages manual FDRs were controlled using the Benjamini‐Hochberg method at an FDR of 5%.

### 2.3 Weighted gene co‐expression network analysis (WGCNA)

WGCNA is a gene co‐expression network‐based strategy for identifying key genes, which is a comprehensive collection of R functions.^21^ WGCNA was based on the expression profiles of lncRNAs and mRNAs from SE and control group. The genes (lncRNAs and mRNAs) with similar expression trend were divided into one module. The screening criteria for selecting genes of WGCNA analysis is *P* value < .05. Then, the GO, KEGG and co‐expression network were analysed and conducted with external software package following the tutorials provided.

### 2.4 Quantitative PCR analysis

The hippocampus was collected for total isolation by TRIzol reagent (Invitrogen) following the instructions of manufacturer. The reverse transcription was performed with the PrimeScript RT Reagent Kit (Perfect Real Time, Takara), following the manufacturer's instructions. Then, the cDNA samples were amplified for qPCR using TB Green Premix Ex TaqTM II (TliRNaseH Plus, Takara). The reaction of qPCR was set at 95°C (30 seconds) for pre‐denaturation, then a total of 40 cycles (95°C for 5 seconds and 58°C for 30 seconds). The relative expression of RNAs was calculated based on the standard curve and *C*_t_ value. The housekeeping gene, β‐actin, was used as a loading control.

### 2.5 Functional annotation and enrichment analysis

Gene ontology enrichment analysis and KEGG enrichment analysis were carried out using the R package named Cluster Profiler based on the differentially expressed RNAs (fold change > 2). The Annotate, Visualize and Integrate Discovery Database (DAVID 6.8) (http://david.abcc. http://ncifcrf.gov/) was used for GO analysis, while the KEGG Oncology‐Based Annotation System 3.0 (KOBAS 3.0) (http://kobas.cbi.pku.edu.cn/) was used for KEGG pathway analysis. Then, the significantly enriched GO or KEGG terms were analysed using hyper geometric test with *P* value ≤ .05.

### 2.6 LncRNA‐miRNA‐mRNA network construction

To construct the lncRNA‐miRNA‐mRNA network, miRNA‐mRNA, miRNA‐lncRNA target relationships were predicted by target prediction database (http://www.targetscan.org/vert_71/and http://www.mirbase.org/). To assess the reliability of candidate ceRNA pairs, filtering strategy is used: (a) adjusted *P* value cut‐off for each ceRNA pairs is set to .01 and (b) circRNA and gene should have the same direction in DEG analysis, because ceRNA pairs were reported to be a positive correlation inexpression. To calculate the probability that a circRNA is a target ceRNA, a Fisher exact test is executed for each pair (circRNA gene) separately. The correlation value cut‐off was 0.90. Cytoscape version 3.6.1 was used for assembly and visualization of the network.

### 2.7 Cell culture and treatment

The rat hippocampal neurons were purchased from JENNIO Bio. Tec. and maintained in a humidified atmosphere (Thermo Fisher) containing 5%CO_2_ at 37°C. The Dulbecco's modified Eagle's medium (DMEM) containing 10% fetal bovine serum (Gibco) was used for neuron cell culture. SiRNA targeting NONRATT010788.2 and siRNA‐NC (RiboBio Tech.) were transfected with fiboFECT CP Transfection kit (RiboBio Tech.) following the manufacturer's protocol. At 48 hours after transfection, the cells were collected for total RNA extraction and related assay. All transfections were performed in triplicate.

### 2.8 Apoptosis detection

At 48 hours after transfection, the cells were collected for apoptosis detection by DeadEnd™ Fluorometric Terminal deoxynucleotidyl transferase deoxyuridine triphosphate nick‐end labelling (TUNEL) system (Promega, WI, USA), following the manufacturer's protocol. The cell nuclei were stained with 4′,6‐diamidino‐2‐phenylindole (Thermo Fisher). The cells with green fluorescence were defined as TUNEL‐positive. The number of TUNEL‐positive cells and total cells in four randomly selected fields was counted. The fluorescence microscope (BX51, Olympus) was used for cell visualization. Annenix V‐FITC/PI dual staining kit (Cat. No. KGA108‐1, KeyGEN Biotech) was employed to detect apoptosis by flow cytometry following the instructions of manufacturers.

### 2.9 Statistical analysis

All of the numerical continuous data were presented as mean ± standard deviation. GraphPad Prism 5.0 was used for statistical analysis, and Student's *t* tests were used for comparing two groups. The *P* value < .05 was considered as statistically significant. All experiments were repeated four to six times.

## 3 RESULTS

### 3.1 SE‐induced changes in expression of lncRNAs in the hippocampus of immature rats

**Figure 1:**
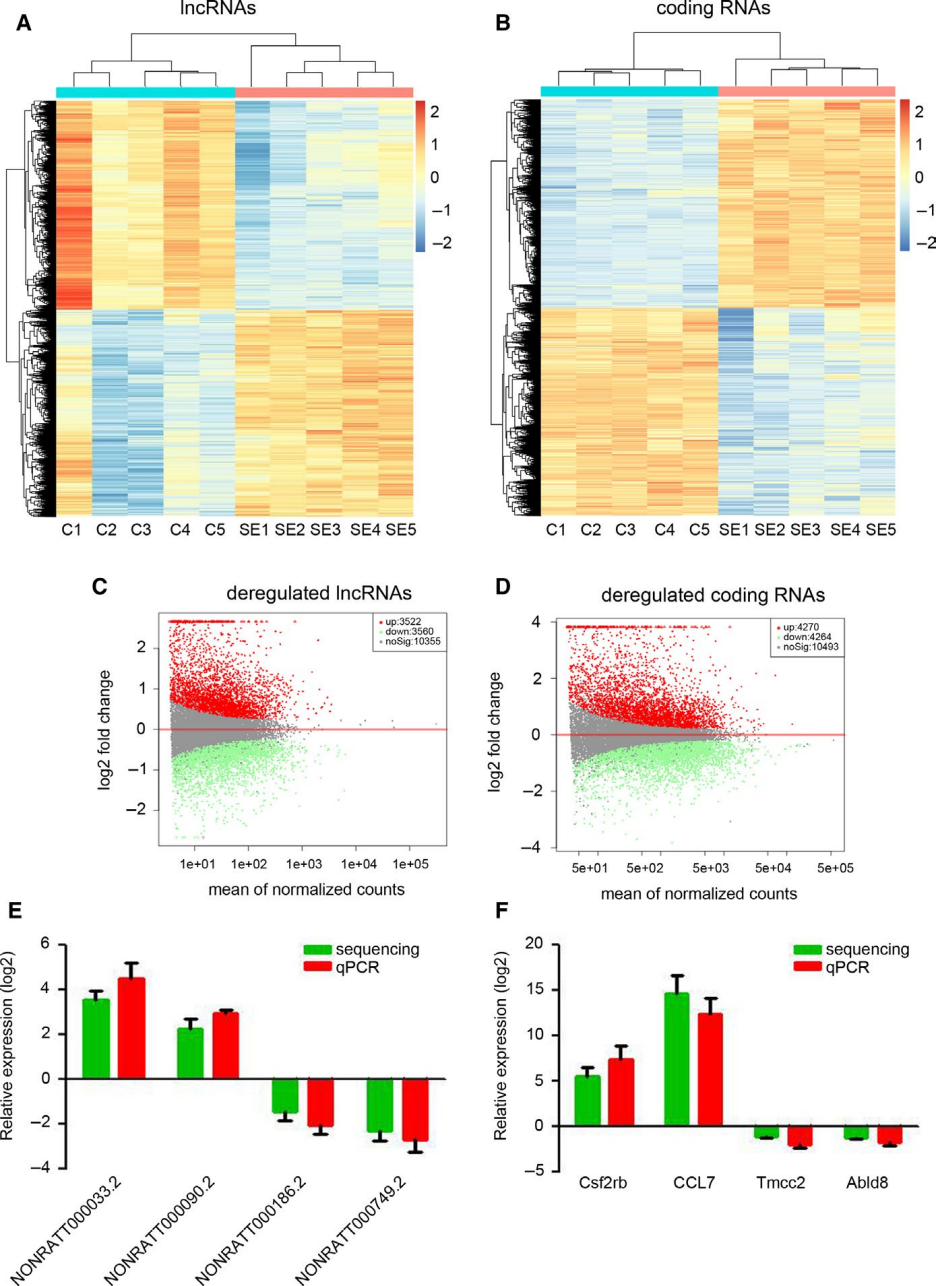
Expression profile of lncRNAs in the hippocampus of immature rats following SE. A and B, The hippocampus of immature rats was collected for high‐throughput sequencing at 24 h post‐SE. Heatmap of differential expression of lncRNA (A) and coding RNAs (B). C and D, Distribution of differentially expressed lncRNAs (A) and coding RNAs. E and F, Comparison analysis of dysregulated lncRNAs (E) and coding RNAs (F) between high‐throughput sequencing and qRT‐PCR results

To investigate the expression profile of lncRNAs during SE development, lithium and pilocarpine were injected to establish the SE model in immature rats. After 24 hours, the hippocampus was collected for high‐throughput sequencing. As shown in Figure 1A and C, 3522 lncRNAs were up‐regulated and 3560 lncRNAs were down‐regulated in the hippocampus of immature rats with SE. Meanwhile, 4270 up‐regulated mRNAs and 4264 down‐regulated mRNAs were identified by high‐throughput sequencing in the hippocampus of SE rats (Figure 1B and D). To validate the accuracy of the high‐throughput sequencing, four random lncRNAs (NONRATT000033.2, NONRATT000090.2, NONRATT000186.2 and NONRATT000749.2) and coding RNAs (Csf2rb, CCL7, Tmcc2 and Abld8) were picked for qPCR determination. Our results indicated that the high‐throughput sequencing results on the expression of selected genes corroborated with those of qPCR (Figure 1E and F). Taken together, we identified the dysregulated lncRNAs and mRNAs in SE.

### 3.2 Potential functional analysis of dysregulated lncRNAS during SE development

**Figure 2:**
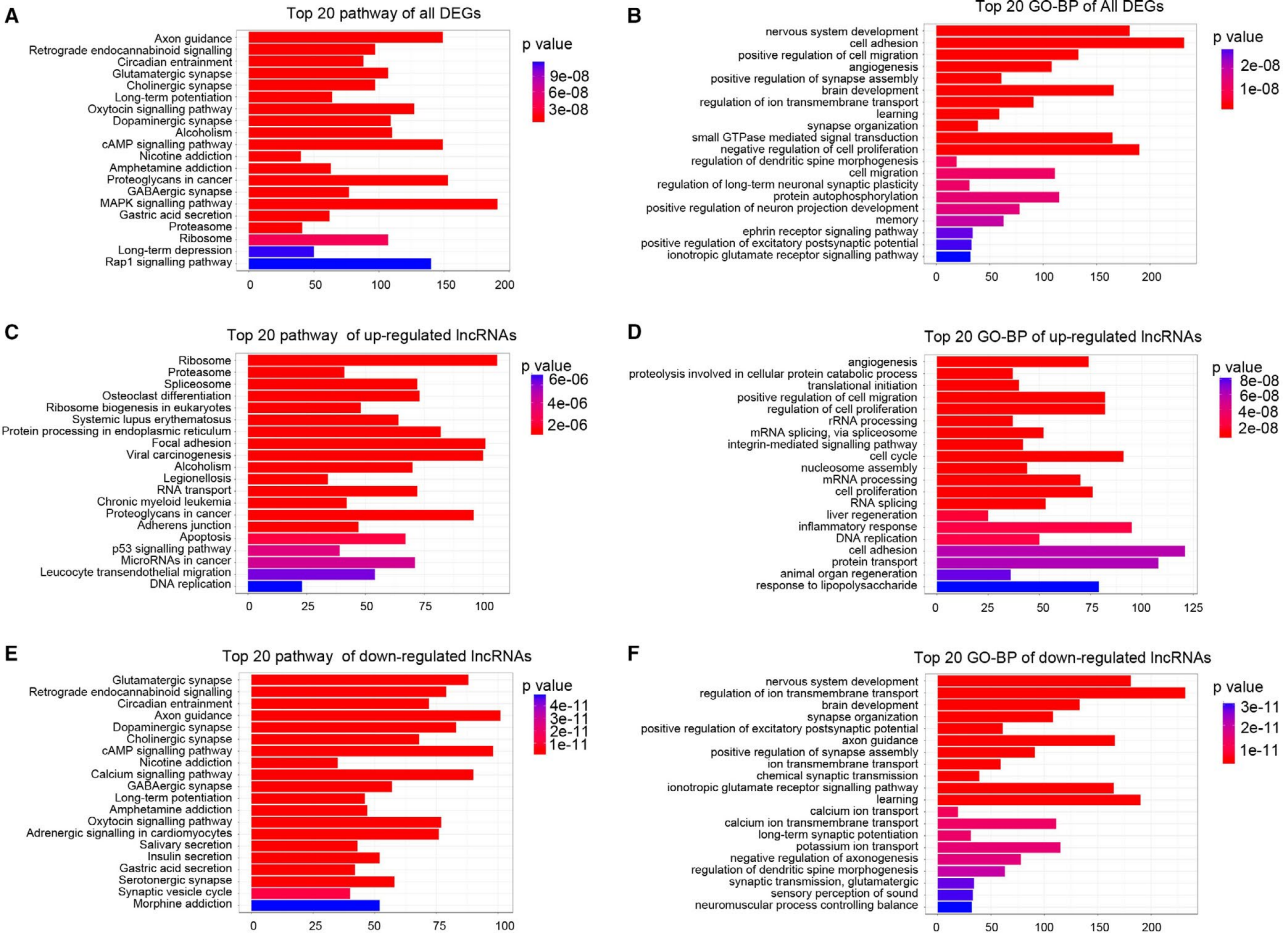
Gene ontology (GO) enrichment and KEGG pathway analysis of differentially expressed genes in the hippocampus of immature rats following SE. A, Top 20 signalling pathways enriched in all dysregulated lncRNAs and coding RNAs. B, Top 20 GO‐BP terms enriched in all dysregulated lncRNAs and coding RNAs. C, Top 20 signalling pathways enriched in up‐regulated lncRNAs and coding RNAs. D, Top 20 GO‐BP terms enriched in up‐regulated lncRNAs and coding RNAs. E, Top 20 signalling pathways enriched in down‐regulated lncRNAs and coding RNAs. F, Top 20 GO‐BP terms enriched in down‐regulated lncRNAs and coding RNAs

Pathway analysis and GO enrichment analysis of differentially expressed lncRNAs are designed to provide insights into the potential functions associated with these genes. Pathway analysis indicated that cGMP signalling pathway, MAPK signalling pathway and Rap1 signalling pathway were regulated by all the dysregulated lncRNAs (Figure 2A). Further results indicated that all the dysregulated lncRNAs were involved in the biological processes of angiogenesis, cell proliferation, cell migration and autophagy (Figure 2B). The up‐regulated lncRNAs were involved in the proteasome, apoptosis and p53 signalling pathways (Figure 2C), while the down‐regulated lncRNAs were involved in the glutamatergic synapse, cAMP signalling, axon guidance and GABAergic synapse pathways (Figure 2E). The biological processes of angiogenesis, cell proliferation, cell cycle and inflammatory response were targeted by up‐regulated lncRNAs (Figure 2D) and the nervous system development, ion transmembrane transport and brain development were targeted by down‐regulated lncRNAs (Figure 2F). These results indicated that the dysregulated lncRNAs may be involved in cell proliferation, inflammatory responses, angiogenesis and autophagy via regulation of cGMP, MAPK and Rap1 signalling pathways.

### 3.3 WGCNA of dysregulated lncRNAS

**Figure 3:**
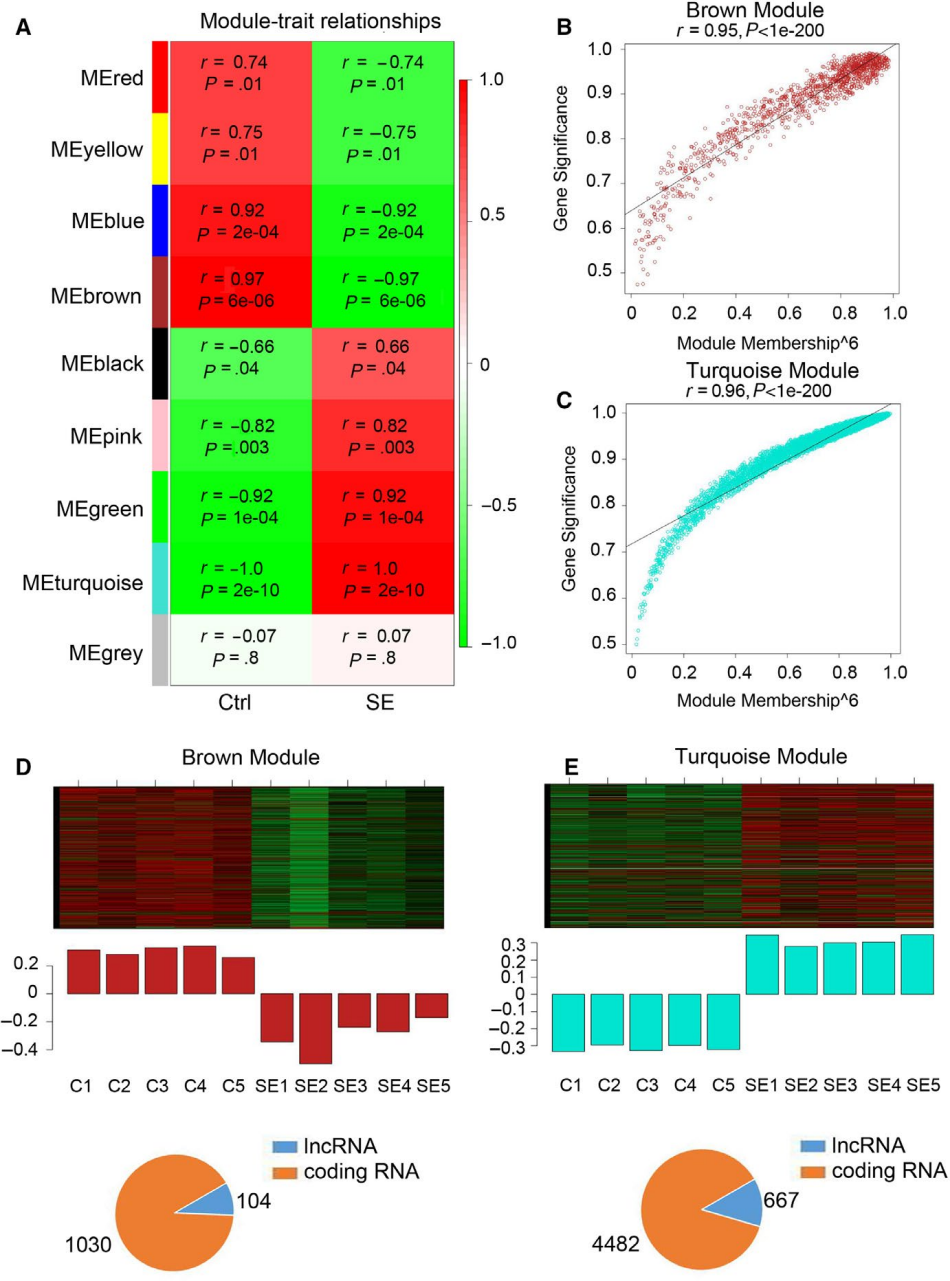
Weighted gene co‐expression network analysis (WGCNA) of differentially expressed genes in the hippocampus of immature rats following SE. A, Specific co‐expression gene modules and their correlation with SE development. Red square indicates a positive correlation, and green square indicates a negative correlation. The r value and *P* value were included in each square. B and C, The gene correlation in brown (B) and turquoise (C) module. D and E, Heatmap in the upper panel is the expression pattern of all genes in the module. The barplot in the middle panel shows the corresponding module gene expression value. The pie chart in the lower panel is the exact number of lncRNAs and coding RNAs in each module

To identify genes expressed together on a higher systems level, all dysregulated lncRNAs and mRNAs were clustered into nine gene modules based on expression trend (Figure 3A). The brown module (*r* = −.97) and turquoise module (*r* = 1.0), which exhibited strongest correlation with the SE, were selected for further analysis. Hub gene analysis implied that most of the dysregulated genes in brown and turquoise modules were the hub genes and were highly correlated with the pathological phenotype of SE (Figure 3B and C). In brown module, 104 lncRNAs and 1030 mRNAs were down‐regulated in the hippocampus of SE rats (Figure 3D). Meanwhile, in the turquoise module, 667 lncRNAs and 4482 mRNAs were up‐regulated in the hippocampus of SE rats (Figure 3E). Taken together, the above results indicated that the lncRNAs in the brown and turquoise modules regulated the pathogenesis of SE.

### 3.4 Potential functional analysis of dysregulated lncRNAS in brown and turquoise modules

**Figure 4:**
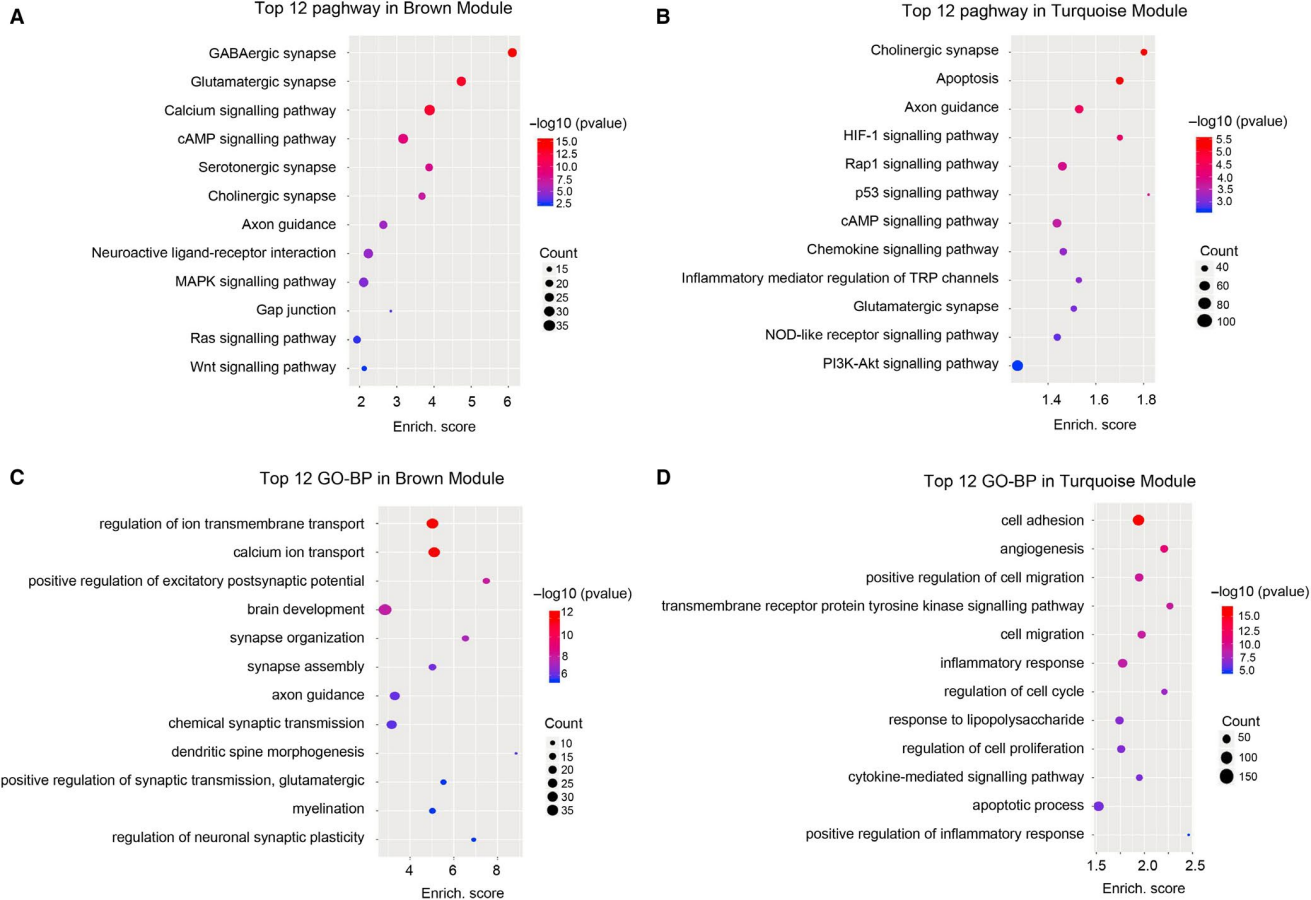
Gene ontology (GO) enrichment and KEGG pathway analysis of dysregulated lncRNAs and coding RNAs in brown and turquoise module. A and B, Top 12 signalling pathways enriched in lncRNAs and coding RNAs of brown (A) and turquoise (B) module. C and D, Top 12 GO‐BP terms enriched in ncRNAs and coding RNAs of brown (C) and turquoise (D) module

To further analyse the potential function of the dysregulated lncRNAs in the brown and turquoise modules, the KEGG and GO‐BP analyses were performed based on the predicted function of the dysregulated genes. Pathway analysis indicated that the brown module was involved in several pathways, including GABAergic synapse, cAMP signalling pathway, MAPK signalling pathway and Ras signalling pathway (Figure 4A), while the apoptosis, Rap1 signalling pathway, cAMP signalling pathway, chemokine signalling pathway, inflammatory mediator regulation of TRP channels and PI3K‐Akt signalling pathway were enriched in the turquoise module (Figure 4B). The highest enriched GO terms targeted by the dysregulated genes in brown module included ion transmembrane transport, brain development and axon guidance (Figure 4C). The biological processes of angiogenesis, cell migration, cell proliferation, inflammatory response and apoptotic process were enriched in turquoise module (Figure 4D). These results indicated that the hub genes in brown and turquoise modules may play a crucial role in the pathogenesis of SE.

### 3.5 Dysregulated lncRNAS regulate SE development by acting as a ceRNA

**Figure 5:**
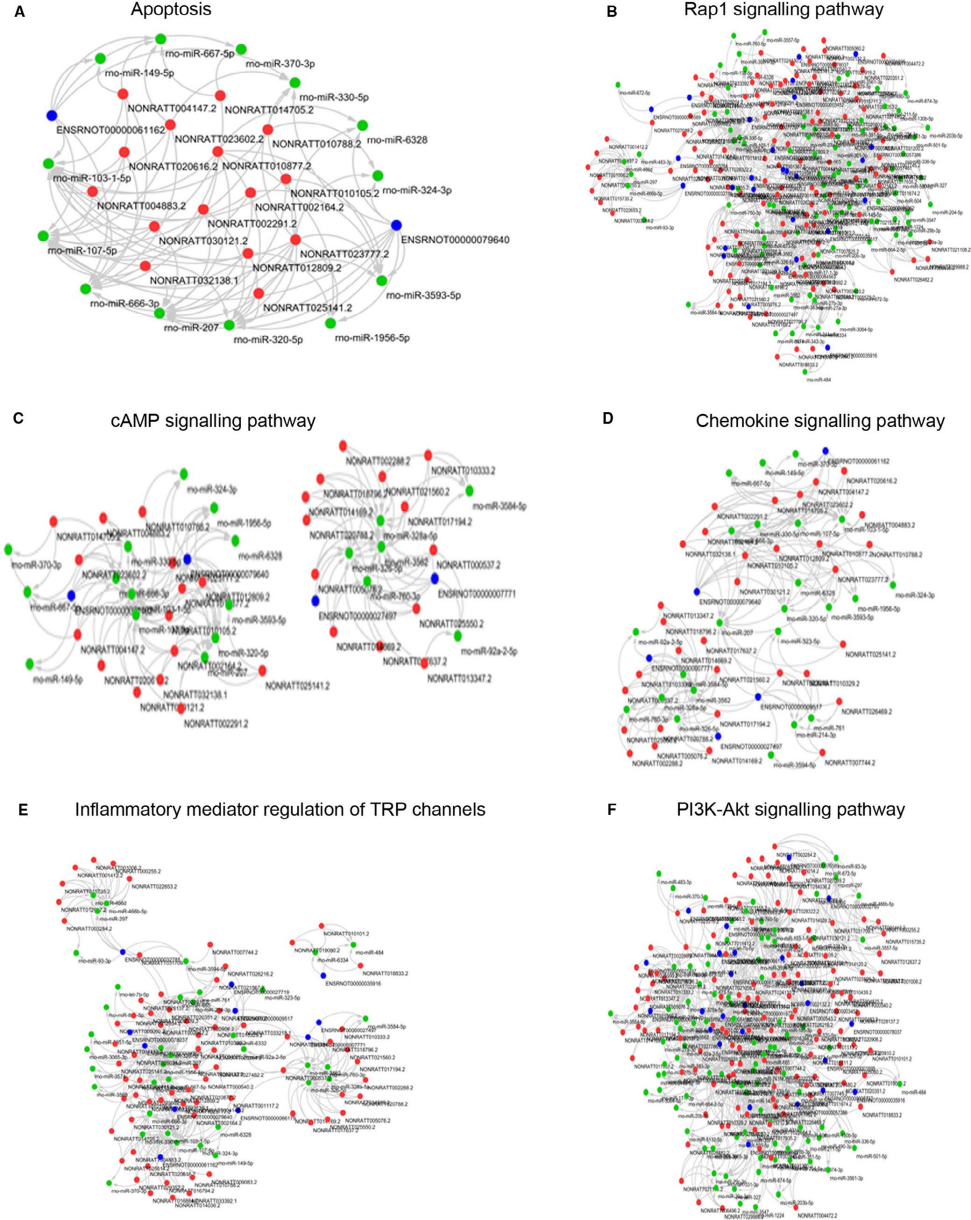
Co‐expression connections of dysregulated lncRNAs and coding RNAs in turquoise module. Co‐expression connections of dysregulated lncRNAs (red), miRNAs (green) and coding RNAs (blue) in the apoptosis (A), Rap1 signalling pathway (B), cAMP signalling pathway (C), Chemokine signalling pathway (D), Inflammatory mediator regulation of TRP channels (E) and PI3K‐Akt signalling pathway (F) of turquoise module

To define the potential ceRNA mechanism under differentially expressed mRNAs and lncRNAs, a ceRNA network was constructed. Thus, we constructed a coding lncRNA‐miRNA‐mRNA co‐expression network based on the miRNA expression profile demonstrated by our previous study.^22^ The network analysis showed that six pathways were regulated by the dysregulated lncRNAs from turquoise module (Figure 5). As shown in Figure 5A, 15 dysregulated lncRNAs were correlated with 13 miRNAs, which were involved in regulating apoptosis. In Rap1 signalling pathway, 121 lncRNAs constructed with 95 dysregulated miRNAs and hence regulated 27 coding RNAs that were demonstrated up‐ or down‐regulated in SE model (Figure 5B). The construction of 28 lncRNAs and 19 miRNAs was participated in cGMP signalling pathway (Figure 5C). In chemokine signalling pathway, 32 lncRNAs were correlated with 23 dysregulated miRNAs (Figure 5D), while 71 lncRNAs that correlated with 37 miRNAs were participated in inflammatory mediator regulation of TRP channels (Figure 5E). The PI3K‐Akt signalling pathway was also regulated by 123 lncRNAs that correlated with 95 miRNAs (Figure 5F). Collectively, these results suggested that dysregulated lncRNAs from turquoise module regulate SE development by acting as a ceRNA.

### 3.6 Knockdown of NONRATT010788.2 inhibits neuronal apoptosis

**Figure 6:**
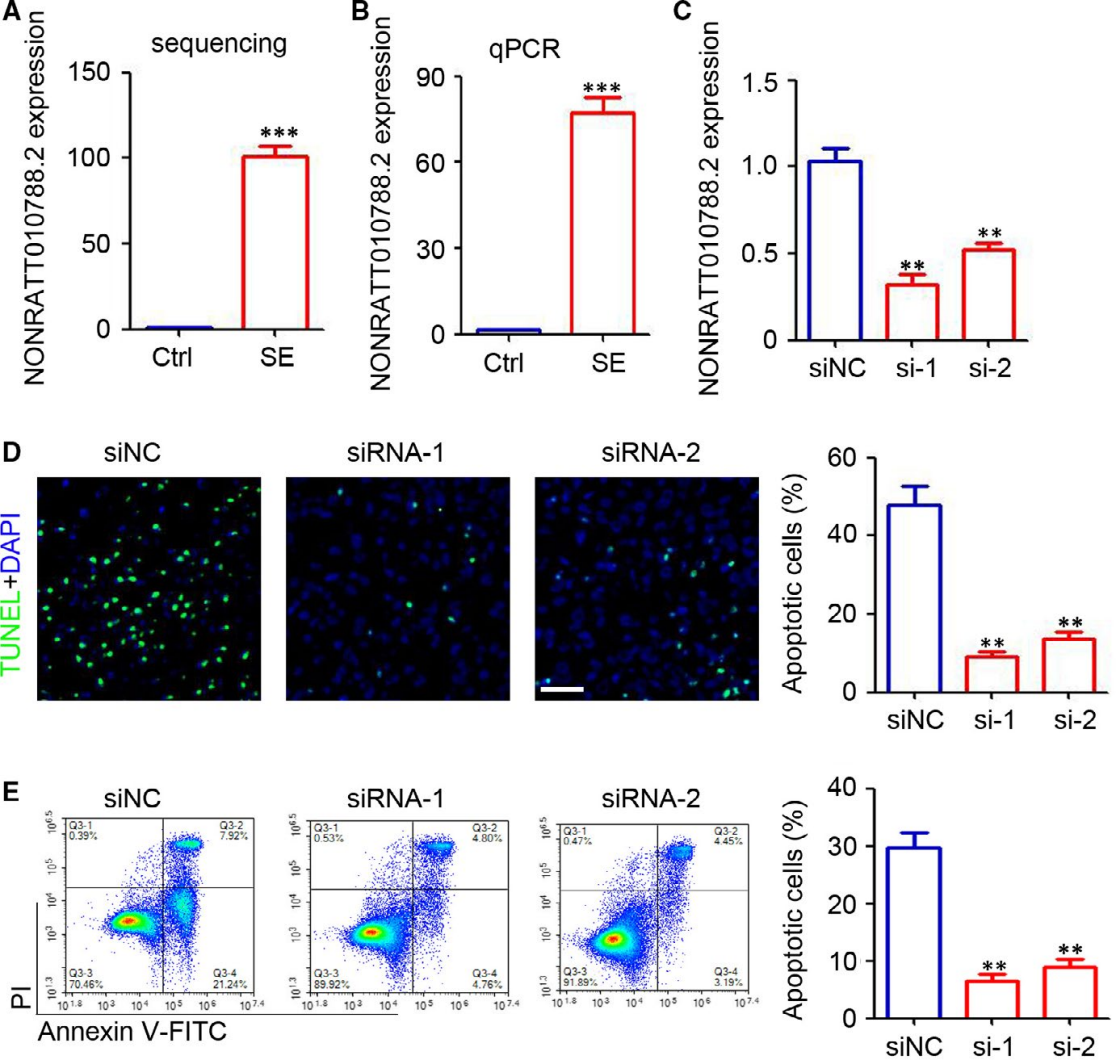
Knockdown of NONRATT010788.2 inhibits neuronal apoptosis. Relative expression of NONRATT010788.2 by high‐throughput sequencing (A) and qRT‐PCR (B) in SE rats and control rats. C, qRT‐PCR analysis of NONRATT010788.2 expression in neurons that transfected with siRNA targeting NONRATT010788.2. D, Detection of apoptosis cells in magnesium‐free medium‐treated neurons that transfected with siRNA targeting NONRATT010788.2 by TUNEL assay. Scale bar = 100 μm. E, Detection of apoptosis cells in magnesium‐free medium‐treated neurons that transfected with siRNA targeting NONRATT010788.2 by Annexin V‐FITC/PI staining. (n = 4, **, *P* < .01; ***, *P* < .0001)

To determine the function of the identified hub lncRNAs, an apoptosis‐related lncRNA (NONRATT010788.2) in turquoise module was selected for further analysis. The high‐throughput sequencing results indicated that NONRATT010788.2 was significantly up‐regulated in the hippocampus of immature rats with SE (Figure 6A). Further, qPCR determination confirmed the up‐regulation of NONRATT010788.2 in the hippocampus of immature rats with SE (Figure 6B). SiRNA targeting NONRATT010788.2 was used to transfect the rat hippocampal neurons, which were treated with Mg^2+^‐free HEPES. Our results confirmed the down‐regulation of NONRATT010788.2 in rat hippocampal neurons transfected with siRNA targeting NONRATT010788.2 (Figure 6C). TUNEL assay indicated that knockdown of NONRATT010788.2 efficiently inhibited the apoptosis of rat hippocampal neurons induced by Mg^2+^‐free HEPES (Figure 6D). Flow cytometry results also confirmed the inhibition of rat hippocampal neuron apoptosis by siRNA targeting NONRATT010788.2 (Figure 6E). Thus, the identified hub lncRNA, NONRATT010788.2, promoted neuronal apoptosis.

## 4 DISCUSSION

LncRNAs may play a crucial role in SE as they are involved in regulation of gene expression at the epigenetic, transcriptional or post‐transcriptional level.^7^, ^8^ In the present study, 7082 lncRNAs (3522 up‐regulated and 3560 down‐regulated) were detected to be dysregulated in the hippocampus of immature rats with SE. Functional prediction indicated that the dysregulated lncRNAs were involved in cell proliferation, inflammatory responses, angiogenesis and autophagy through regulation of cGMP, MAPK and Rap1 signalling pathways. WGCNA identified 667 up‐regulated hub lncRNAs in turquoise module involved in apoptosis, inflammatory responses and angiogenesis via regulation of HIF‐1, p53 and chemokine signalling pathways and via inflammatory mediator regulation of TRP channels. LncRNA‐miRNA‐mRNA construction indicated that dysregulated lncRNAs from turquoise module regulate SE development by acting as a ceRNA. Knockdown of an identified hub lncRNA (NONRATT010788.2) inhibits neuronal apoptosis in vitro. The dysregulated lncRNAs are potential therapy and diagnosis targets for SE.

Understanding the expression profile and function of coding genes and non‐coding genes in SE would expand the understanding of the pathogenesis of SE and provide novel therapeutic target for SE. In a previous study,^22^ 29 up‐regulated and 20 down‐regulated miRNAs were identified in developing rat hippocampi. Another study reported that overexpression of miR‐96 significantly repressed brain damage in SE rats by inhibiting Atg7 and Atg16L1 expression and autophagosome formation in the hippocampus.^20^ Previous studies also have indicated that lncRNAs, H19 and UCA1, may be involved in the pathogenesis of SE via regulation of immune and inflammatory responses, cell apoptosis, activation of MAPK and NF‐κB signalling pathway.^17^, ^18^, ^19^ However, no study has been performed to investigate the expression profile of lncRNAs in SE. Our study first demonstrated the expression profile of lncRNAs in the hippocampus based on a Lithium/Pilocarpine‐induced SE model in immature rats. Further functional prediction indicated that the dysregulated lncRNAs were involved in cell proliferation, inflammatory responses, angiogenesis and autophagy, which have been demonstrated to play an important role in the pathogenesis of SE.^23^, ^24^, ^25^, ^26^ Meanwhile, a coding lncRNA‐miRNA‐mRNA co‐expression network was constructed based on the miRNA expression profile demonstrated by our previous study^22^ and indicated that dysregulated lncRNAs were participated in various signalling pathways that involved in SE development.^27^, ^28^

WGCNA is an R software package which can be used to search for clusters (modules) of highly correlated genes, for summarizing an intramodular hub gene, for weighted correlation network analysis, for example co‐expression network analysis of gene expression data.^21^ Based on the WGCNA, the module with a high correlation with the disease progression would be screened out.^29^, ^30^ The dysregulated genes in the module are usually defined as hub genes that are involved in the pathogenesis of disease.^29^, ^30^ In the present study, the differentially expressed lncRNAs and mRNAs were divided into nine modules. There was a strong correlation between SE and two modules (brown module and turquoise module). Further functional prediction and lncRNA‐miRNA‐mRNA co‐expression network analysis indicated that the up‐regulated hub lncRNAs in turquoise module were involved in SE progression through regulation of apoptosis, Rap1 signalling pathway, cAMP signalling pathway, chemokine signalling pathway, inflammatory mediator regulation of TRP channels and PI3K‐Akt signalling pathway, which have been demonstrated to play an important role in the pathogenesis of SE.^31^, ^32^, ^33^, ^34^, ^35^ Furthermore, the in vitro model was studied to confirm the functional role of an identified hub lncRNA (NONRATT010788.2) that was associated with apoptosis. The constructed co‐expression network indicated that lncRNA, NONRATT010788.2, inversely correlated with miR‐324‐3p, which plays a negative role in apoptosis.^36^, ^37^ It is indicated that NONRATT010788.2 may bind to miR‐324‐3p and hence regulate neuronal apoptosis. These results confirmed the promotional role of NONRATT010788.2 in neuronal apoptosis, but further investigations are needed to demonstrate the underlying mechanism.

In conclusion, our study is the first to demonstrate the expression profile and potential function of lncRNAs in the hippocampus of immature rats with SE. The defined hub lncRNAs may participate in SE development through regulation of apoptosis, inflammatory responses and angiogenesis in the hippocampus of immature rats. Thus, the dysregulated lncRNAs would be the potential therapy and diagnosis targets for SE. But, further experimental studies are needed to investigate the functions of these hub lncRNAs in SE, as well as the underlying mechanism.

## CONFLICT OF INTEREST

The authors declare that they have no potential conflicts of interest.

## AUTHORS’ CONTRIBUTIONS

J G and Ly H conducted all the experiments. Dz M and Y Q participated in the design of the study and helped to draft the manuscript. R L conducted the statistical analysis. Qy C and Fy Z participated in the physiological examination. Dz M and J G designed the project and finalized the manuscript. All authors read and approved the final manuscript.

## FUNDING INFORMATION

This work was supported by the National Natural Science Foundation of China (No. 81501301, 81971433, 81971428, 81330016, 81630038, 81771634, 81842011), the grants from Science and Technology Bureau of Sichuan Province (2016TD0002) and the grant of Clinical Discipline Program (neonatology) from the Ministry of Health of China (1311200003303).

## ETHICAL APPROVAL

All experimental procedures were approved by the Institutional Animal Care and Use Committees of Sichuan University.

## CONSENT FOR PUBLICATION

Consent for publication is not applicable in this study. No individual person's data were used.

## Data Availability

Not applicable.
